# How accurately is ncRNA aligned within whole-genome multiple alignments?

**DOI:** 10.1186/1471-2105-8-417

**Published:** 2007-10-26

**Authors:** Adrienne X Wang, Walter L Ruzzo, Martin Tompa

**Affiliations:** 1 Department of Computer Science and Engineering, University of Washington, Box 352350, Seattle, WA 98195, USA; 2 Department of Genome Sciences, University of Washington, Box 355065, Seattle, WA 98195, USA

## Abstract

**Background:**

Multiple alignment of homologous DNA sequences is of great interest to biologists since it provides a window into evolutionary processes. At present, the accuracy of whole-genome multiple alignments, particularly in noncoding regions, has not been thoroughly evaluated.

**Results:**

We evaluate the alignment accuracy of certain noncoding regions using noncoding RNA alignments from Rfam as a reference. We inspect the MULTIZ 17-vertebrate alignment from the UCSC Genome Browser for all the human sequences in the Rfam seed alignments. In particular, we find 638 instances of chimeric and partial alignments to human noncoding RNA elements, of which at least 225 can be improved by straightforward means. As a byproduct of our procedure, we predict many novel instances of known ncRNA families that are suggested by the alignment.

**Conclusion:**

MULTIZ does a fairly accurate job of aligning these genomes in these difficult regions. However, our experiments indicate that better alignments exist in some regions.

## Background

In this time when so many genome sequences are reaching completion, alignments of multiple whole genomes are of great value to biologists, since enlightening evolutionary information is encoded in the conservation and variation across species. Multiple alignment on genomic scales is also a great challenge to algorithm designers. Protein-coding regions usually evolve more slowly than noncoding regions, and therefore tend to be easier to align. In contrast, noncoding regions are still challenging to align correctly. Because of this, a number of recent reviews and articles [1-4] have made compelling pleas for methods to assess the accuracy of multiple sequence alignments and to compare the alignments produced by different tools.

We use alignments of noncoding RNA (ncRNA) as a test of the accuracy of multiple alignment of genomic regions that are difficult to align. This is a rather challenging test, as many functional RNAs exhibit weak primary sequence conservation [5]. As a byproduct of our procedure, we predict many novel instances of known ncRNA families that are suggested by the alignment. We return to this topic in the Discussion section.

### Evaluating the accuracy of multiple sequence alignments

Multiple sequence alignment is a difficult computational problem. Technically, the problem of finding an optimal multiple sequence alignment is *NP*-complete [6]. In practice, what this means is that finding an optimal multiple sequence alignment requires computation time that grows exponentially with the number of sequences to be aligned, making it impractical for aligning more than a few sequences. Note that this is true even if the sequences are relatively short; aligning multiple genome-size sequences adds another level of complexity.

Because of this inherent difficulty, algorithm designers are forced to invent heuristic methods that produce results that can only approximate the optimal multiple sequence alignment. Many such alignment tools have been proposed over the past 20 years. Since they employ various heuristics intended to approximate an optimal solution, any two such methods often produce incomparable results when aligning the same set of input sequences. This situation naturally raises the questions of (1) assessing the quality of resulting multiple alignments and (2) deciding which of several multiple alignments produced by different tools is "best".

The situation becomes even worse when we consider whole-genome multiple alignments. This is due to the multitude of genomic rearrangements (inversions, translocations, duplications, chromosome fusions and fissions, etc.) that occur over the course of evolution [7]. The traditional alignment problem is not defined to deal with such "nonlinear" events. In what follows, we will categorize methods that have been proposed for assessing the accuracy of traditional multiple sequence alignments (where much work has been done), and highlight those that have been applied to assess the accuracy of whole-genome multiple alignments (where relatively little has been done to date).

1. One approach to measuring the accuracy of multiple sequence alignments is to use artificial sequences resulting from a simulation of evolutionary processes [8-11]. Since the experimenter can track all evolutionary events, identifying the truly homologous characters is straightforward. These known homologies can be used to measure the accuracy of any alignment to be tested. This approach has also been employed to evaluate genome-size multiple alignments by Blanchette *et al*. [12] and by Prakash and Tompa [13]. An obvious drawback of this simulation approach is its sensitivity to assumptions about the underlying evolutionary processes, which are not at all well understood.

2. Another approach is to run the alignment program on a set of sequences in which certain features are known *a priori *to be homologous, and measure the accuracy with which these known homologous features are aligned. This approach was used in genome-size alignment studies by Brudno *et al*. [14] and by Margulies *et al*. [3]. The most obvious choice for the known homologous features is a set of coding exons. However, this choice suffers from the shortcoming that such features are usually well conserved and easy to align, and most algorithms do so quite accurately. In addition to using coding exons, Margulies *et al*. [3] also tested alignment accuracy using ancestral repeats, which tend to be more challenging to align correctly than coding exons. For this general approach, though, many known sets of homologous sequences have been discovered using some alignment algorithm, which leads to circularity if then used to test the accuracy of an alignment algorithm. In addition, no accuracy information is provided for aligned regions other than the previously known orthologous features.

3. A number of papers have suggested methods that inspect multiple sequence alignments, judging regions of the alignment that show good conservation across the aligned sequences to be well aligned, and even removing sequences from the alignment that show lack of good conservation [15-18]. While good conservation often implies good alignment, the converse need not be true. In fact, in our case of interest, the alignment of homologous RNA elements, it is well known that sequence conservation may be very low if instead the RNA secondary structure conservation is strong [19].

4. Lassmann and Sonnhammer [20] proposed a measure to assess alignment quality by comparing several multiple sequence alignments, assuming that regions identically aligned by multiple tools are more reliable than regions differently aligned. This method requires several auxiliary alignments in order to evaluate the alignment of interest. In the same general class are methods that employ other auxiliary information, such as protein secondary structure, in order to assess alignment quality [21].

5. A final approach is one designed to provide a statistical assessment of the accuracy of a given multiple sequence alignment, by extending the theory of Karlin and Altschul [22] from pairwise to multiple alignments. This was done for short multiple alignments by Prakash and Tompa [23], and later extended by them to measure the alignment quality of all portions of a genome-size multiple sequence alignment [13]. In the latter paper the authors found approximately 10% of the UCSC Genome Browser's human chromosome 1 alignment to be suspiciously aligned. This is a portion of the same whole-genome alignment we analyze in this paper.

### RNA families as a test of accuracy

The approach we take falls into the second category listed above. Instead of using well conserved coding regions as the homologous test features, however, we use curated alignments of noncoding RNA (ncRNA) from Rfam (version 7.0, March 2005). Rfam is a collection of ncRNA families that contains multiple sequence alignments and covariance models of these families [24,25].

**Figure 1:**
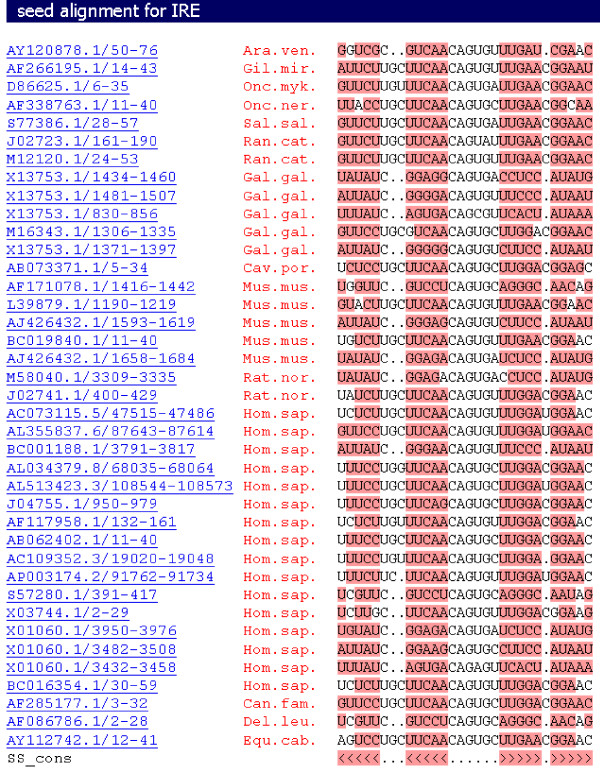
**Seed alignment of the IRE (Iron Response Element family, RF00037)**. The first column provides the accession numbers of the sequences. The species can be found in the second column. The third column is the alignment. The last row in the seed alignment shows the predicted secondary structure of this family. The color blocks represent predicted base paired regions.

As an illustration, Figure 1 shows Rfam's seed alignment and the predicted secondary structure of the Iron Response Element family (RF00037). Most functional RNAs tend to maintain their base-paired structure over conservation of primary sequence [19]. To illustrate this, in Figure 1, the primary sequences of the 10th element (Gal.gal. X13753.1/830-856) and the 11th element (Gal.gal. M16343.1/1306-1335) are different. For example, the 10th element has a U in the first position, and an A in the last position, while the 11th element has a G and a C respectively. However, this does not disrupt the similarity of their predicted secondary structures, because both UA and GC form Watson-Crick base pairs.

The seed alignment of each Rfam family is hand-curated by human experts, based on both the secondary structure and primary sequence of trusted example RNAs. Using the Infernal software package [26], the seed alignment is used to train a covariance model [24,25], a statistical model akin to a hidden Markov model. Together with a score threshold also chosen by the Rfam curators, these models are then used to search genomic DNA for additional instances of the specific RNA family represented by the seed alignment, automatically producing Rfam's so-called full alignments. We use Rfam's covariance model and score threshold as the definitive rule for membership in ncRNA families and for alignment. Rfam and Infernal are considered by experts to be the most accurate general tools for defining membership in ncRNA families and for alignment of family members. There are several databases of recognized ncRNAs of various types but, to the best of our knowledge, only Rfam/Infernal provides both expert-curated alignments and a simple automated search tool leveraging those alignments.

Multiple sequence alignments considering the secondary structure such as Rfam's covariance models are more reliable than alignment tools that only consider primary sequences. This makes these seed alignments a challenging test case for any whole-genome multiple alignment that is based on primary sequence alone. We apply this challenging test to the 17-vertebrate whole-genome alignment produced by MULTIZ [12] and available on the UCSC Genome Browser [27]. Although MULTIZ is most frequently shown to be quite accurate in these challenging cases, our study does reveal occasional compelling evidence of misalignment. Two of the more interesting categories are summarized here:

1. For 5.4% of the nonhuman sequences aligned to some human ncRNA, what is aligned to the human ncRNA is not a contiguous sequence. Instead, it is composed of sequence fragments from different regions or even different chromosomes, none of these fragments individually passing Infernal's test of ncRNA family membership. We call these "chimeric alignments". For 45% of these chimeric alignments, one of the aligned fragments can be extended in its native genomic context to reveal a full ncRNA family member that is probably the correct alignable ortholog. These improvable chimeric alignments are compelling instances of misalignment.

2. For 5.1% of the nonhuman sequences aligned to some human ncRNA, what is aligned includes a large gap in the nonhuman species, and the aligned fragment by itself again does not pass Infernal's threshold for ncRNA family membership. We call these "partial alignments". For 26% of these partial alignments, the fragment can be extended in its native genomic context to reveal a full ncRNA family member that is probably the correct alignable ortholog. These improvable partial alignments are compelling cases of false negative orthology predictions by the alignment algorithm.

### RNA prediction from reliable alignments

Two groups have developed RNA prediction tools using comparative genomics methods [28,29]. The MULTIZ whole-genome alignment of 8 vertebrate species was used, and the segments with low phastCons [30] sequence conservation scores and segments with too few species were removed. This filtering process leaves a set of conserved segments spanning less than 5% of the reference human genome. Based on the conserved segments, they evaluated structural conservation of base-pairing patterns and identified tens of thousands of candidate functional RNA elements. Our goal is opposite to the goal of these groups. They assumed the MULTIZ multiple sequence alignment to be reliable, and used that to predict ncRNAs. We assume that known ncRNA covariance models provide reliable alignments, and use them to evaluate the accuracy of the MULTIZ multiple alignments in those difficult noncoding regions. Torarinsson *et al*. [31] discovered many conserved RNA structures in regions that MULTIZ did not align at all, but are presumably syntenic.

### UCSC genome browser and MULTIZ

The UCSC Genome Browser is a powerful web tool that provides the reference sequence and working draft assemblies for a large collection of genomes [27]. It is a multifaceted and reliable display of any requested portion of genomes at any scale, as well as many annotation tracks, including assembly contigs and gaps, mRNA and expressed sequence tag alignments, multiple gene predictions, cross-species homologies, single nucleotide polymorphisms, sequence-tagged sites, radiation hybrid data, transposon repeats, and more as a stack of coregistered tracks.

The 17-vertebrate whole-genome alignment that we evaluate is taken from the UCSC Genome Browser database. The 17 vertebrates of the alignment are human (hg), chimp (panTro), rhesus monkey (rheMac), rat (rn), mouse (mm), rabbit (oryCun), cow (bosTau), dog (canFam), armadillo (dasNov), elephant (loxAfr), tenrec (echTel), opossum (monDom), chicken (galGal), frog (xenTro), tetraodon (tetnig), fugu (fr), and zebrafish (danRer). Some species have not been fully sequenced or have low sequence coverage. The 17-vertebrate whole-genome alignment (to human genome assembly NCBI Build 36.1, UCSC hg18, March 2006) is an update and expansion of the older 8-vertebrate alignment. It was produced by a computational pipeline including BLASTZ [32], chaining and netting [33], and MULTIZ [12]. For the remainder of this paper, we will refer to this computational pipeline simply as "MULTIZ". MULTIZ uses the progressive alignment technique to align multiple sequences, including highly rearranged or incomplete sequences.

## Results

We first extract all the human sequences in the seed alignments from the Rfam database [24,25]. These sequences are then uploaded to the UCSC genome browser [27] to find the chromosome number, strand, and start and end positions of the sequences in the human genome using the sequence alignment tool BLAT [34]. After we get the precise locations of the sequences in the human genome, we retrieve MULTIZ multiple alignment files with each human Rfam sequence as the reference sequence.

**Figure 2:**
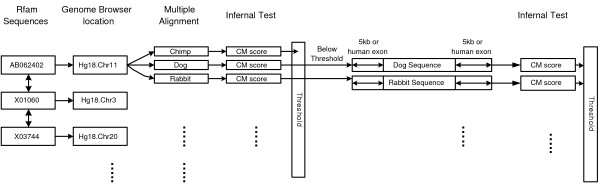
Overview of the first phase of the evaluation process.

**Figure 3:**
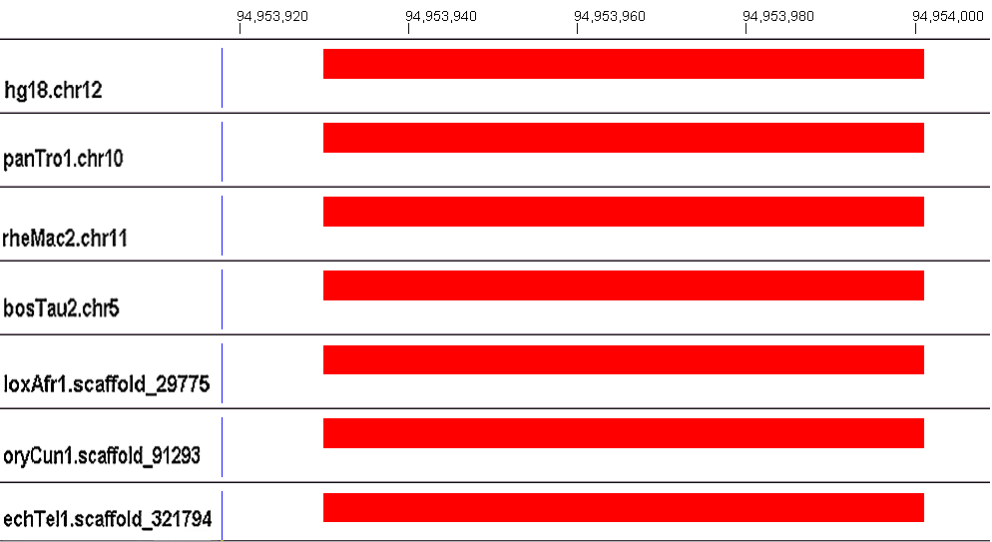
**A perfect alignment**. This figure illustrates a perfect alignment of a human tRNA (RF00005) on chromosome 12. Red rectangles denote sequences with high covariance model scores in all figures. See Methods for further explanation of these figures.

We use the software package Infernal (version 0.7) to analyze each of the sequences aligned by MULTIZ to the human ncRNA. Infernal uses covariance models for sequence analysis of ncRNA families [26]. For each ncRNA family, a covariance model based on the seed alignment and a threshold score are provided by Rfam. The seed alignment and threshold are curated by experts. Any sequence for which Infernal assigns a covariance model score above the threshold is classified as a member of that family. If a sequence aligned by MULTIZ to the human ncRNA element returns a score above the threshold, we conclude that this alignment is supported by Rfam. Otherwise, we search the nearby region of the MULTIZ alignment to look for a family member. The search space is bounded by 5 Kb on each side of the human element or the distance to the nearest human exon on each side, whichever is less. The motivation for this search is that, if a local misalignment has occurred, the orthologous ncRNA family member may be in the neighborhood of its aligned family members. We detect such an orthologous family member by using Infernal to search the neighborhood for any segment with a score above the threshold provided by Rfam. We find the bound of 5 Kb to be sufficiently liberal: of all the family members found within a 5 Kb neighborhood, 90% are within 10 bp of the nearest human family member, and only 8 instances (1%) are more than 100 bp from the nearest human family member. Figure 2 is an overview of this first phase of the evaluation process. The first phase evaluation is done on all seed alignments in Rfam, and the results are summarized in Table 1. We extract 585 human elements from 201 ncRNA families. Of these, 172 human elements are perfectly aligned with members of the same RNA family in every aligned species (see Figure 3). These are labeled "perfect alignments" in Table 1. The remaining 413 imperfect alignments have at least one aligned sequence receiving poor covariance model score, and require a neighborhood search to the nearest human exons or the 5 Kb boundary. These are not necessarily misalignments: many may indicate loss of an ncRNA in some species.

**Table 1:** Statistics of alignments to human ncRNAs in seed alignments

	Count	Percent
Perfect alignments	172	29.4%
Imperfect alignments	413	70.6%

Human ncRNAs in seed alignments	585	100%

The count indicates the number of human ncRNA elements and their associated multispecies alignments.

We find 424 human ncRNAs in the neighborhood searches of the 413 imperfect alignments. The 11 additional human ncRNAs are elements not included in the seed alignments, but identified by Infernal to be members of the same ncRNA family. There are 6043 segments in other species aligned to human ncRNAs, including the perfect alignments in Table 1. Table 2 categorizes these as follows:

**Table 2:** Statistics of aligned segments in nonhuman species

	Count	Percent	Improvable	Percent
Perfectly aligned elements	4025	66.61%	-	-
Shifted elements	707	11.70%	-	-
Chimeric alignments	328	5.43%	146	44.5%
Partial alignments	310	5.10%	79	25.5%
Possible alignment inversions	4	0.06%	-	-
Possible losses of ncRNA	669	11.10%	-	-

Aligned segments in nonhuman species	6043	100%	-	-

The count indicates the number of segments in other species aligned to (or near) a human ncRNA. The last two columns show the number of cases of alignments that can be improved by straightforward realignment (see text).

**Figure 4:**
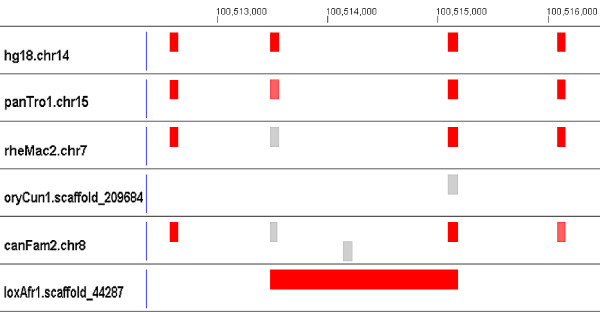
**A shifted element**. This figure illustrates a case of large shift of an element aligned to human SNORD113 (C/D box small nucleolar RNA SNORD113/SNORD114, RF00181) on chromosome 14. A single RNA in elephant (loxAfr1.scaffold 44287) is divided into halves by the alignment, shown here as the very long red rectangle, with the left half aligned to the beginning of the second of the four human RF00181 elements shown, and the right half to the right end of the third human instance. In the MULTIZ alignment, this appears as though it were a 1556 bp deletion in elephant, spanning from the middle of the second human instance to the middle of the third.

**Figure 5:**
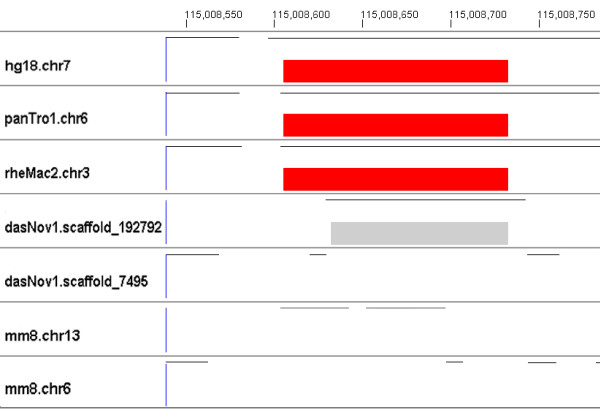
**Chimeric alignments**. This figure illustrates two examples of chimeric alignments of a human SNORA25 (small nucleolar RNA SNORA25, RF00402) on chromosome 7. a. Two pieces of sequences from two different scaffolds (dasNov1.scaffold 192792 and dasNov1.scaffold 7495, the two rows directly below the red rectangles) in armadillo are concatenated and aligned to the human ncRNA. However, when we extract a longer sequence from the genome at the position of the first fragment, dasNov1.scaffold 192792, the aligned fragment can be extended to a member of the small nucleolar RNA SNORA25 family. b. Two pieces of sequences from two different chromosomes in mouse (mm8.chr13 and mm8.chr6, the next two rows below armadillo) are aligned to the same human ncRNA. The first fragment, if extended, is also a member in the family. Note that neither armadillo nor mouse show a red rectangle, since these chimeric alignments score below the covariance model threshold. The thin horizontal lines show which regions of that species are included in the alignment.

**Figure 6:**
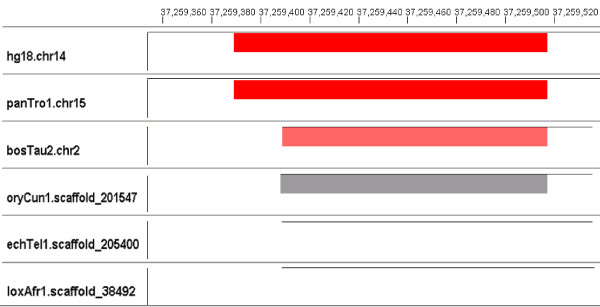
**Partial alignments**. This figure illustrates partial alignments of a human SNORA42 (small nucleolar RNA SNORA42, RF00406) on chromosome 14. The segments in rabbit (oryCun1.scaffold 201547), tenrec (echTel1.scaffold 205400) and elephant (loxAfr1.scaffold 38492) appear to be partially aligned to the human ncRNA. These segments all receive scores above the threshold for this family if extended. Note that the partially aligned sequences score below the threshold, as indicated by the absence of red rectangles. Note also that the cow sequence in this alignment (bosTau2.chr2) is categorized as a shifted element in Table 2. The thin horizontal lines show which regions of that species are included in the alignment.

• 4025 RNA family members in other species are perfectly aligned to human ncRNAs; that is, the segment aligned to the human ncRNA is assigned a high covariance model score without any neighborhood search. The rest of the aligned segments receive low covariance model score.

• For 707 segments, an Rfam member that is not perfectly aligned to other human ncRNAs in the family can be found in the neighborhood search. These are labeled as shifted elements in Table 2. Figure 4 illustrates a case of a particularly large shift in an alignment of a human SNORD113 (C/D box small nucleolar RNA SNORD113/SNORD114, RF00181) on chromosome 14. An RNA element in elephant (loxAfr1.scaffold 44287) is divided into halves by the alignment, each half aligned to a different human ncRNA. The two human ncRNAs are 1556 bp apart. Most of the 707 shifts are very small compared to this example.

• For some species, the portion aligned to a single human ncRNA is not a contiguous sequence. Instead, it is composed of sequence fragments from different regions or even different chromosomes. These 328 discontiguous segments are labeled as chimeric alignments in Table 2. Figure 5 illustrates two examples of chimeric alignments of a human SNORA25 (small nucleolar RNA SNORA25, RF00402) on chromosome 7. Two sequences from different regions (dasNov1.scaffold 192792 and dasNov1.scaffold 7495) in armadillo are concatenated and aligned to a single human ncRNA. Also, two fragments from different chromosomes in mouse (mm8.chr13 and mm8.chr6) are aligned to the single human ncRNA.

• Some of the segments receive low score because that species is only partially aligned to the human ncRNA. We find 310 such partial alignments. In this case, the segment might be a family member if the sequence were fully aligned in that region. Figure 6 illustrates partial alignments of a human SNORA42 (small nucleolar RNA SNORA42, RF00406) on chromosome 14. The segments in rabbit (oryCun1.scaffold 201547), tenrec (echTel1.scaffold 205400) and elephant (loxAfr1.scaffold 38492) appear to be partially aligned to the human ncRNA.

• 4 segments appear to be possible alignment inversions: a family member is identified on the other strand, but otherwise perfectly aligned to the human family member. These are all tRNAs in opossum. Since the number is so small, we will omit this category from the remaining discussion.

• 669 low score segments, fully aligned and contiguous, do not reveal a family member in the neighborhood search regions. These may well indicate actual loss of an ncRNA in that species rather than misalignment. Note that this case does not include the many instances when a species is completely absent from the alignment to the human ncRNA.

We perform a second phase of evaluation of each partial alignment. For each of the 310 partial alignments that receives a low covariance model score, we extract from its genome the original aligned segment plus 200 bp upstream and 200 bp downstream. (Note that this contextual sequence may not even be aligned by MULTIZ.) We use Infernal to search for a family member in this contiguous 400+ bp sequence that includes the fragment MULTIZ had aligned to the human ncRNA. In 79 of the 310 cases, we succeed in finding such a family member. As an example, in Figure 6, the segments in rabbit (oryCun1.scaffold 201547), tenrec (echTel1.scaffold 205400) and elephant (loxAfr1.scaffold 38492) all receive scores above the threshold of this family if extended.

A similar process is applied to each of the 328 chimeric alignments we find in the first phase of evaluation. We extract from its genome the chimeric fragments aligned to human ncRNAs, plus 200 bp upstream and 200 bp downstream of each fragment. This results in at least two 400+ bp sequences. In total, we find that family members could be restored in 146 of the 328 chimeric alignments by this simple process. For example, in Figure 5, for the species armadillo, when we extract a longer sequence from the genome at the position of the first fragment, dasNov1.scaffold 192792, this fragment extends to become a family member. For mouse, the extended sequence of the fragment in mm8.chr13 is also a member in the family.

**Figure 7:**
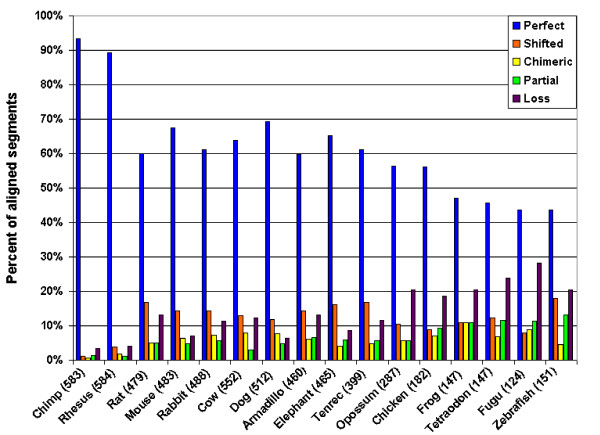
**Categorization of aligned segments by species**. For each species, the bars show the percent of aligned segments for that species in each of the categories of Table 2. The number next to the species name is the number of alignments in which that species is included.

We next categorize the aligned segments of Table 2 by species. The results are shown in Figure 7. For each species, the bars show the percent of aligned segments for that species in each of the five categories of Table 2 (omitting inversions because of the small number). The number next to the species name is the number of alignments in which that species is included.

## Discussion

MULTIZ does a fairly accurate job of aligning the vertebrate genomes in the ncRNA regions, particularly given the challenging nature of correctly aligning these elements. 66.6% of the elements aligned by MULTIZ to human ncRNA have a high covariance model score. These are very likely to be true positives of the alignment method. Conversely, only the large shifts, the 328 chimeric alignments, and the 79 improvable partial alignments are strong candidates as false positives and false negatives of the alignment method. These total approximately 7% of the elements aligned by MULTIZ to human ncRNA. Our results also suggest ways to improve some of the imperfect alignments.

Our results are roughly consistent with those of Prakash and Tompa [13], who used entirely different methods to assess whole-genome alignment accuracy, based on the statistical theory of Karlin and Altschul [22]. Like us, they found that MULTIZ generally was quite accurate at aligning orthologous sequences, but they also identified approximately 10% of MULTIZ's human chromosome 1 alignment to be suspiciously aligned.

Comparing our findings to those of Brudno *et al*. [14] on the 1 Mb CFTR region, their multiple alignment tool MLAGAN perfectly aligned at least 94% of the human coding exons to the orthologous exons in each of 8 other mammalian genomes. This gives support to the belief that ncRNA elements are more difficult targets for accurate multiple alignment than coding exons are. Margulies *et al*. [3] performed similar experiments on the ENCODE regions, but reported much lower accuracy in aligning coding exons for the four multiple alignment algorithms they tested, TBA, MLAGAN, MAVID, and PECAN. Possible explanations for their lower accuracy include the following:

1. The CFTR region is nicely syntenic, with few duplications, in the aligned species, whereas most of the ENCODE regions are not as well conserved.

2. The quality of exon annotation in the ENCODE regions was not as high as that in the CFTR region, which has received more attention.

3. Some species had not yet been sequenced in a particular ENCODE region, or its sequence was not chosen in ENCODE's orthology prediction phase.

4. The MLAGAN alignment of the CFTR region was of 12 species, whereas the ENCODE alignments were of 28 species, which is more challenging.

If the procedures we described for testing ncRNA alignment accuracy were to be repeated for whole-genome alignments produced by other tools such as MLAGAN, MAVID, and PECAN, we predict that the results would be comparable to what we reported in Table 2 and Figure 7. We do not believe any of our results to be peculiar to MULTIZ. Instead, we believe that they illustrate an inherent difficulty in aligning ncRNA for any alignment method that relies on primary sequence alone. However, Margulies *et al*. [3] reported a surprising level of discrepancy among the four alignments they tested, with MAVID generally demonstrating somewhat lower sensitivity and specificity than that of the other methods, so we would certainly expect some similar amount of variance in the accuracy of ncRNA alignment.

Our assessment of alignment accuracy is only as good as Infernal's predictions. If the covariance model is inaccurate in certain instances, our assessment may be inaccurate as well. However, since the covariance model incorporates secondary structure, we trust that it is a reliable guide to the correct alignment of ncRNA.

In 11.7% of the aligned segments that we inspect, we find that the nearest family member in the other species is shifted from the human element. Most of these shifts seem rather small, and so represent minor misalignment or may not represent misalignment at all. For instance, 90% of these 707 shifted elements have both ends within 10 bp of the aligned human family member. It is worth noting, however, that even small misalignments may have adverse effects on some downstream analyses.

Our experiments reveal that in 5.4% of the cases, MULTIZ aligns discontiguous fragments to one human ncRNA, and it is likely to be quite misleading to draw any biological inferences from such chimeric alignments. Further analysis suggests that 44.5% of the chimeric alignments could be improved to family members by extending one of the fragments and aligning the extended piece to the human element. These improvable chimeric alignments are compelling instances of misalignment.

We also observe 5.1% of the cases to be partial alignments. These partial alignments fail to reveal a complete aligned RNA element. 25.5% of these partial alignments can be improved to family members by extending the aligned fragment. These improvable partial alignments are compelling cases of false negative orthology predictions by the alignment algorithm.

Washietl *et al*. [29] and Pedersen *et al*. [28] both use the MULTIZ whole-genome alignment to predict RNA secondary structures. The accuracy of their tools largely depends on the accuracy of the multiple alignment. Their results may be subject to false negative predictions due to various types of misalignments described above. Focusing on the highly conserved regions identified by the phastCons method, as they did, may have reduced the incidence of these misalignments since the regions where MULTIZ is likely to align imperfectly are often regions that are not highly conserved. On the other hand, such a strict selection likely causes them to miss many interesting candidates [31] while providing incomplete protection against alignment errors: phastCons was never intended as a method for measuring alignment correctness. Indeed, its purpose is to measure conservation, assuming that the alignment correctly aligns orthologous sites. Prakash and Tompa [13] have shown that misalignment of one sequence can often be found even in regions where phastCons scores are very high due to strong conservation in the remaining sequences.

There are 11.1% of the alignments that receive low covariance model score, but we do not find any better candidate in the neighborhood of the alignment. We do not have enough evidence to classify this type of alignment. It could be caused by loss of ncRNA element in that species, failure of the covariance model (e.g., an excessively restrictive family model or score threshold), or misalignment by MULTIZ.

Looking at Figure 7, there are some fairly clear trends in the alignments as we move farther from human in the phylogeny. The primates, not surprisingly, are extremely well aligned to human. The remaining mammals have a very similar breakdown to each other according to the five categories of alignment (with the possible exception of opossum, which looks more like chicken). Somewhat surprisingly, there seem to be no qualitative differences distinguishing the low-coverage assemblies rabbit, armadillo, elephant, and tenrec from the other nonprimate mammals. The remaining, more distantly related vertebrates show a decrease in the percent of perfectly aligned segments and a commensurate increase in the percent of partial alignments and possible losses of ncRNA as we move farther from human.

Many of the vertebrate instances of ncRNA elements discovered by our procedure are not included in Rfam. In theory, the Rfam staff could find all these instances (and many more) by running Infernal on each of the vertebrate genomes individually, ignoring alignment. However, it currently requires approximately one month using 1000 computers to approximate this Infernal search on their 8 Gb database. It is clearly impractical to extend this to all 17 sequenced vertebrates, and the gap is rapidly widening because of newly sequenced genomes. As an alternative, we propose using whole-genome alignment and lists of human ncRNAs from Rfam, restricting Infernal's search for family members to the sequences aligned to the human ncRNAs and their immediate neighborhoods. Although not exhaustive, our procedure provides an efficient and effective method for predicting ncRNA family members in newly sequenced genomes. By using whole-genome alignments in conjunction with Infernal as described, we exploit orthology and synteny with two good effects: (1) the search is made efficient by limiting it to the most promising regions suggested by the alignment and (2) the alignment adds extra evidence to Infernal's predictions, likely decreasing the incidence of false positives.

## Methods

### Visualization tool

All alignments and Infernal results are available for viewing [35] using GMAJ [36], which was used to produce the images in Figures 3, 4, 5, 6. GMAJ is a tool for viewing and manipulating multiple sequence alignments. It offers a coloring feature for both the interactive graphical and text representation of the alignments. We use this feature to color all ncRNA elements, as well as the regions aligned to human ncRNA elements. The regions with a covariance model score above and within ten bits of the threshold are colored pink. Regions that receive a covariance model score above the threshold plus ten bits are colored red. Regions with a covariance model score below and within ten bits of the threshold are colored dark gray. Regions with a covariance model score below the threshold minus 10 bits but above 0 are colored light gray. The first row displays tick marks corresponding to the positions in the human reference sequence.

## Authors' contributions

AXW wrote all the programs and conducted the experiments. All authors participated in the design of the study, and read and approved the final manuscript.
